# Ontogeny of Social Relations and Coalition Formation in Common Ravens (*Corvus corax*)

**Published:** 2012

**Authors:** Matthias-Claudio Loretto, Orlaith N. Fraser, Thomas Bugnyar

**Affiliations:** University of Vienna, Austria; Konrad Lorenz Forschungstelle, Austria; University of Vienna, Austria; University of Vienna, Austria; Konrad Lorenz Forschungstelle, Austria

**Keywords:** International Journal of Comparative Psychology, Behavior, Behaviour, Communication, Vocalization, Comparative Psychology, Behavioral Taxonomy, Behavioural Taoxonomy, Cognition, Cognitive Processes, Intelligence, Ravens, Language, Social Intelligence

## Abstract

The social intelligence hypothesis, originally developed for primates to explain their high intelligence and large relative brain size, assumes that challenges posed by social life in complex societies with many group members lead to the evolution of advanced cognitive abilities. In birds, pair-bonded species have larger brains than non-pair bonded species, indicating that the quality of social relationships better predicts social complexity than group size. Ravens are a long-term monogamous and territorial species, renowned for their sophisticated socio-cognitive skills and complex social relationships. Notably, during their early years they live in fission-fusion-like non-breeder societies in which social relationships could be of particular importance. Here we observed the development of dominance and affiliative relationships in 12 hand-raised captive ravens, examining the influence of age, sex and kinship on social interactions. Furthermore, we investigated at which developmental step a stable hierarchy emerged, whether third-party interventions played a role and how selectively birds intervened in others’ conflicts. At 4-5 months post-fledging, we found an increase in socio-positive behaviour and a decrease in aggression, along with the establishment of a linear dominance rank hierarchy. In line with kin selection theory, siblings exhibited a greater degree of tolerance and engaged in more socio-positive behaviour. In their first few months, ravens frequently intervened in others’ conflicts but supported mainly the aggressor; later on, their support became more selective towards kin and close social partners. These findings indicate that ravens engage in sophisticated social behaviours and form stable relationships already in their first year of life.

The social intelligence hypothesis (Humphrey, 1976; Jolly, 1966) or Machiavellian mind (Byrne & Whiten, 1988) assumes that challenges posed by social life lead to evolution of advanced cognitive abilities. In support of this Sawaguchi and Kudo (1990) and Dunbar (1992) found that mean group size (as proxy for social complexity) correlates with relative brain size in primates. The same pattern could be found for carnivores and insectivores (Dunbar & Bever, 1998) and cetaceans (Connor, 2007; Marino, 2002). In addition, more direct measures for socio-cognitive skills, such as tactical deception (Byrne, 1996) and social play (Lewis, 2000) correlate with relative brain size in primates. However, such relationships do not hold for mammals in general, e.g., ungulates (Shultz & Dunbar, 2006), and notably not for birds; instead, in those taxa, long-term monogamy appears to be more important as pair-bonded species have larger brains than non-pair bonded species (Dunbar & Shultz, 2007; Emery, Seed, Von Bayern, & Clayton, 2007). This indicates that quality and kind of social relationships are probably better predictors for social complexity than group size (Emery et al., 2007).

Most populations of group-living vertebrates are structured by dominance and affiliative relationships (de Waal & Tyack, 2003). Dominance hierarchies can be clearly established and follow either a transitive (e.g., A > B > C and A > C) or nonlinear (when the rank order is irregular or circular e.g., A > B > C and C > A) order (Martin & Bateson, 1993). Linear dominance hierarchies are generally stable over time as long as the composition of the group does not change (Senar, Camerino, & Metcalfe, 1990). Affiliative relationships can consist of kinship (i.e., affiliates that share genes), partnership (i.e., affiliates that engage in reproduction) and “friendships” (i.e., affiliates that are non-kin and non-mates).

In many groups, variation in relationship quality is a good predictor for subjects’ social behaviour. Notably, individuals with valuable relationships are more likely to provide help during or after conflicts, form coalitions and alliances, share resources and/or information (Cords, 1997; Silk, 2002; van Schaik & Aureli, 2000). Theoretically, there are two other components of social relationship quality in addition to value (which refers to the immediate benefits afforded by the relationship), namely the compatibility of the relationship, which refers to the general tenor of social interactions within the dyad, and the security of a relationship, representing the consistency or predictability of interactions over time (Cords & Aureli, 2000). The existence of these three components of relationship quality has recently been confirmed empirically in chimpanzees (Fraser, Schino, & Aureli, 2008) and in ravens (Fraser & Bugnyar, 2010), indicating that social bonds may work similarly in mammals and birds.

From a cognitive point of view, primates are renowned for their ability to differentiate between familiar and unfamiliar individuals (Cheney & Seyfarth, 1990) and even to categorize group members hierarchically according to their dominance rank and kinship (Bergman, Beehner, Cheney, & Seyfarth, 2003). They thus handle not only their own relationships with others (dyadic level), but appear to be capable of representing relationships among others (triadic level) (Cheney & Seyfarth, 1986, 1989; Judge, 1991; Judge & Mullen, 2005; Sinha, 1998). There is also some indication of third-party understanding in non-primates (rooks, Emery et al., 2007; e.g., hyenas, Engh, Siebert, Greenberg, & Holekamp, 2005; pinyon jays, Paz-Y-Miño, Bond, Kamil, & Balda, 2004). However, the evidence is sometimes indirect and patterns can be difficult to interpret. In fact, interactions involving third-parties do not necessarily afford an understanding of their relationships; they may be based on simpler mechanisms, such as following rules of thumb (Engh et al., 2005).

Studies on the understanding of social relationships in birds are underrepresented (Emery et al., 2007), which is surprising since some groups such as corvids show striking cognitive abilities both in the physical and social domain (review in Emery, 2006). A possible reason could be that most species do not live in stable groups, but are characterised by a relatively high degree of fission-fusion dynamics, with the pair-bond being the main stable unit (Emery et al., 2007; but see Scheiber et al., 2008). However, many species show seasonal differences in sociality (they are more solitary during the breeding season) or may vary social tendencies across developmental periods. Specifically, large-brained birds like corvids tend to have a prolonged period of social maturation, spending extensive time with their parents and/or in non-breeder groups (Haffer, 1993; Ratcliff, 1997). Unfortunately, little is known about the development of social relationships during this time.

We here investigated the ontogeny of social relationships in common ravens (*Corvus corax*). This species is renowned for their sophisticated socio-cognitive skills such as tactical deception (Bugnyar & Kotrschal, 2002), gaze following, perspective taking (Bugnyar, Stöwe, & Heinrich, 2004; Schloegl, Kotrschal, & Bugnyar, 2007), and possibly even knowledge attribution (Bugnyar & Heinrich, 2005). This contradicts with their apparently relatively ‘simple’ social life as long-term monogamous and territorial adults. However, ravens often do not become reproductively successful until their fifth year of life, in some cases delaying reproduction until their 10^th^ year (wild ravens; T. Bugnyar, unpublished data), indicating one of the longest periods of socio-cognitive development of any avian species (Fraser & Bugnyar, 2010). After becoming independent from their parents at about 6 months of age, juvenile ravens regularly join non-breeder groups for foraging but also for roosting and socializing (Haffer, 1993; Ratcliff, 1997). During this phase, ravens in captivity (Gwinner, 1964) as well as in the wild show a dominance hierarchy in competition for valuable resources (Huber, 1991). Captive ravens have also been described to form strong affiliative relationships (Gwinner, 1964), whereby kin in particular appear to share valuable relationships and female-female relationships are less stable and more insecure than those of males and between males and females (Fraser & Bugnyar, 2010).

The objective of this study was to observe the development of young ravens’ dominance and affiliative relationships. In the first step, we examined whether the birds’ social interactions were affected by age (developmental period), sex and kinship. We then investigated (i) when a stable hierarchy emerged, (ii) the role of third-party interventions in achieving and maintaining dominance rank and (iii) the birds’ selectivity in intervening in others’ conflicts. We expected aggression to decline over time and, conversely, affiliation to increase over time. Interventions in conflicts could affect acquisition of dominance rank through aggressor support and/or the maintenance of rank through selective support of particular individuals. In the latter case, selectivity in support during conflicts should be linked to affiliation patterns observed outside conflicts.

## Method

### Study Subjects

In Spring 2004 we hand-raised 13 ravens (7 males, 6 females) at the Konrad Lorenz Forschungsstelle in Grünau, Austria. Ravens from two nests (containing 4 and 3 birds respectively) were zoo-bred, ravens of the two other nests (containing 3 birds each, one nest of three siblings, one nest with three non-siblings) were taken from the wild with permission of the Ministerium für Landwirtschaft, Umweltschutz und Raumordnung des Landes Brandenburg, Germany. The nests were physically, but not visually, separated in an indoor aviary. Before fledging we transferred all birds into one big nest in an outdoor aviary (approximately 240m^2^), situated in the Cumberland Wildpark in Grünau, Austria. The aviary contained trees, branches, tree trunks, stones and shallow pools for bathing. Once fledged, we kept the birds together in one social group with two unrelated adult ravens (a 4-year-old female and an 8-year-old male). The adult birds were excluded from data analysis as well as one young female, which showed atypical social behaviour due to severe illness during the nestling phase. This female was taken out of the group together with one (affiliated) male in August. Another two young males were predated by a marten at the age of eight months. For individual recognition, birds were marked with coloured leg-rings. They were fed twice per day (in the morning and evening) with meat, milk products and kitchen leftovers. Water was provided *ad libitum*.

### Data Collection

Data were collected by M.L. over the course of a 12 month period (May 2004-April 2005). Protocols were taken before the morning or evening feedings using focal and behavioural sampling techniques (Martin & Bateson 1993). Focal observations (5 min per bird) were conducted on a daily basis for the first two months post-fledging but were then reduced to approximately one week (4-6 days) per month; altogether, this resulted in 752 focal protocols. In addition, behavioural observations on the entire group (i.e., all subjects at the same time, for a total of 30 min) were carried out throughout the study period, resulting in a total of 61 ad-lib-protocols. In either case, all affiliate and agonistic social interactions among study subjects were recorded. We defined affiliate social interactions as contact sitting (sitting within one body’s length of a partner), preening (preening a partner with the beak) and touching (briefly touching any part of a partner’s body with the beak). Agonistic interactions included retreat (approached bird moves away), forced retreat (approached bird retreats after being threatened), submission (approached bird stays but shows submissive behaviour), threat back (approached bird stays and threatens back), and fight (jumping at, hitting with the beak and/or chasing another bird).

For each interaction, we recorded the subject (initiator) and partner (recipient) identities, the type of interaction, and the response. Furthermore, we recorded whether a third party (i.e., individual originally not involved in the conflict) joined an ongoing agonistic interaction by threatening or physically hitting one of the combatants; these cases were termed agonistic support.

### Data Analysis

As many of the eight behavioural categories we recorded were likely to correlate with each other, we used principal component analysis (PCA) to reduce the number of individual behavioural variables to a few composite behavioural dimensions (Budaev, 2010; Tabachnick & Fidell, 2007). All frequencies of individual behaviours were calculated per observation minute, hence for these analyses we could also include those individuals that were not present for the entire observation period (in total *N* = 12). We used cube-root transformations to improve normality for each of the variables. We calculated the correlation matrix with a sample size of *N* = 132. The sampling adequacy was appropriate (Bartlett’s sphericity test χ^2^ = 525.64, *df* = 28, *p* < 0.001; KMO = 0.750). To extract factors, we used a minimum eigenvalue of 1.0. A varimax rotation, an orthogonal rotation method that minimizes the number of variables that have high loadings on each component, was used to simplify the interpretation of the components. Coefficients of correlation were considered as high loadings when greater than 0.5 or less than −0.5. The extracted components are by definition uncorrelated with each other. The total variance explained is the sum of the variance explained by each extracted component.

Using linear mixed models (LMMs) we assessed the effects of different developmental periods of the first year post-fledging, kinship and sex combination on the components obtained by the PCA (dependent variables). For each dependent variable, we added together the frequencies of all variables that had high loadings on that component in the PCA, and split the data into each of three developmental periods (period 1: May-August; period 2: September-December; period 3: January-April). Kin were defined as siblings, which were hand-raised together in one nest until fledging (*N* = 26 kin dyads out of all possible dyads *N* = 132). In all models, subject and partner identity were included as random factors to control for between-subject variation and non-independence of data points. The best model was selected by using Akaike’s information criterion (AIC), which compares the adequacy of several models and identifies the model that best explains the variance of the dependent variable as that with the lowest AIC value (Burnham & Anderson, 2004; Tabachnick & Fidell, 2007). We present only the effects of those variables that occur in the best model. An alpha level of 0.05 was adopted for all tests. Pair wise comparisons were corrected with sequential Bonferroni procedure (Holm, 1979). The PCA and the LMMs were conducted in SPSS version 17.0 (SPSS Inc., Chicago, IL, U.S.A.).

In the following analyses, we only used the 9 individuals that were present all time from May 2004 until April 2005 during all observations. We tested the presence of a linear dominance hierarchy separately for each period (May-August, September-December, January-April) using MatMan 1.1 (de Vries, 1995; de Vries, Netto, & Hanegraaf, 1993) by analyzing matrices of forced retreat interactions. Because of unknown relationships between dyads, instead of using Landau’s linearity index h (Landau, 1951; Martin & Bateson, 1993), we used the improved linearity index h’ (de Vries, 1995). To assess the statistical significance of the degree of linearity a two-step randomization test (10000 randomizations) was performed (de Vries, 1995). Analysis of hierarchy characteristics included a calculation of the directional consistency (DC) index: the total number of times the behaviour was performed in the main direction within each dyad minus the number of times the behaviour occurred in the less frequent direction within each dyad divided by the total number of times the behaviour was performed. It ranges between 0 (completely equal exchange) and 1 (completely unidirectional) (van Hooff & Wensing, 1987).

To test for correlation between agonistic support and preening, we created a square symmetrical matrix for both interaction frequencies and assessed the correlation by means of the Mantel test (Schnell, Watt, & Douglas, 1985). To obtain the significance of Mantel’s Z, we performed a permutation procedure with 10000 permutations (Jackson & Somers, 1989). Furthermore, the dual normalization test described in Freeman, Freeman, & Romney (1992) was used to correct for individual variation. All calculations concerning dominance hierarchy or matrix correlations were performed using MatMan 1.1 (de Vries et al., 1993). Additionally, we investigated differences in frequency of agonistic support over periods using the Friedman test and posthoc Wilcoxon signed-ranks tests with sequential Bonferroni correction. Finally, we looked for selectivity of support for aggressors or kin. To do this, we supplemented our data set with additional ad libitum data on agonistic support collected by colleagues during the study period in order to maximise our sample size.

## Results

### Behavioural Components

From our eight observed behavioural variables, three components were extracted by the PCA. Components 1, 2 and 3 explain 37.4%, 29.7%, and 13.2% of overall variance respectively, totalling 80.3%. See Table 1 for loadings for each of the behavioural variables on each extracted component. Each variable loaded strongly onto a single component. The first extracted component included high loadings from the behavioural variables retreat, forced retreat, defensive behaviour and threat back. All of these behaviours show a lack of tolerance toward another individual and represent a weak form of aggression, and so we labelled this variable “intolerance.” Contact sitting, preening and touching had high loadings on the second component, which we labelled “socio-positive behaviour.” The third extracted component was characterised by high loadings of fighting and therefore represented severe “aggression.”

### Factors Influencing the Components

**Figure 1:**
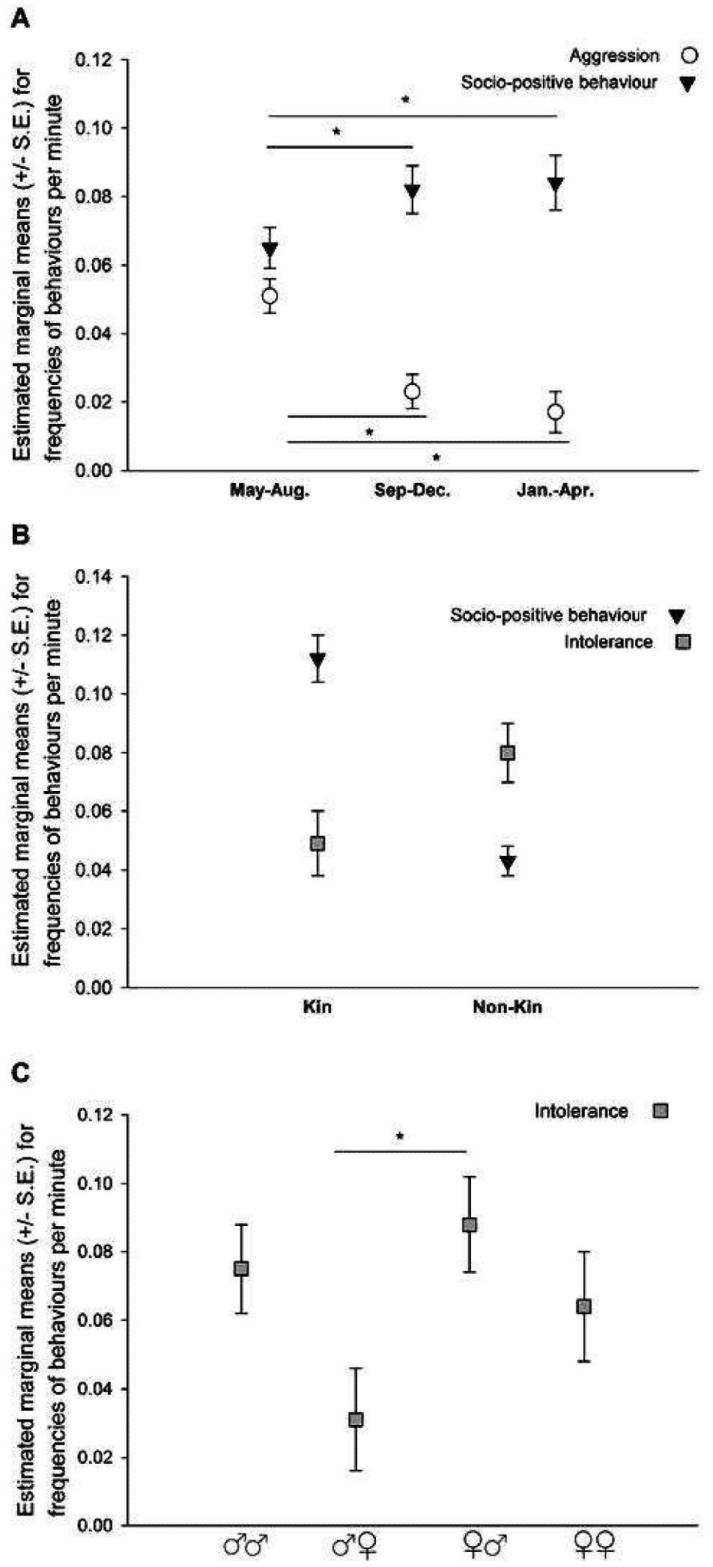
The effects of developmental period (**A**) and kinship (**B**) on mean individual frequencies of sociopositive (circles) and aggressive (triangles) behaviour, and of sex-combination (**C**) on intolerance (squares). *p* < 0.05 (after Bonferroni corrections)

Socio-positive behaviour was significantly influenced by period (LMM: *F*(2, 310.13) = 4.232, *p* = 0.015, Figure 1A) and kinship, *F*(1, 307.20) = 95.077, *p* < 0.001, Figure 1B. Between kin, socio-positive behaviour was significantly more frequent than between non-kin. Pair-wise comparisons between periods showed a significantly lower frequency of socio-positive behaviour in the first than in the second or third period (period 1 vs. 2: *df* = 306.914, *p* = 0.015, period 1 vs. 3: *df* = 313.744, *p* = 0.016, period 2 vs. 3: *df* = 309.238, *p* = 0.761, Figure 1A).

Intolerance was significantly affected by kinship, *F*(1, 297.37) = 23.888, *p* < 0.001, Figure 1B, and sex combination, *F*(3, 31.69) = 4.258, *p* = 0.012, but not by period. Siblings showed a significantly lower frequency of intolerance (i.e., they were more tolerant of each other). Pair-wise comparisons between sex combinations after Bonferroni correction showed that females were significantly less tolerant of males, than males were of females (*df* = 30.649, *p* = 0.006, Figure 1C).

Severe aggression was only significantly affected by developmental period, *F*(2, 304.91) = 44.585, *p* < 0.001. Pair wise comparisons revealed that the frequency of severe aggression in the first period was significantly higher than in the second or third periods (period 1 vs. 2: *df* = 303.611, *p* < 0.001, period 1 vs. 3: *df* = 308.447, *p* < 0.001, period 2 vs. 3*: df* = 302.095, *p* = 0.152, Figure 1A).

### Linear Dominance Hierarchy

For the first period we could not reject the null hypothesis of randomly distributed dominance relationships (*h’* = 0.517, *χ^2^* = 29.76, *df* = 20.16, *p* = 0.078, *DC* = 0.662), however, for period 2 and 3 we could accept the alternative hypothesis of a linear ordering of dominance relationships (period 2: *h’* = 0.667, *χ^2^* = 36.56, *df* = 20.16, *p* = 0.015, *DC* = 0.903, period 3: *h’* = 0.725, *χ^2^* = 37.76, *df* = 20.16, *p* = 0.015, *DC* = 0.920). Starting from November all males outranked all females.

### Agonistic Support by Third Party

**Figure 2:**
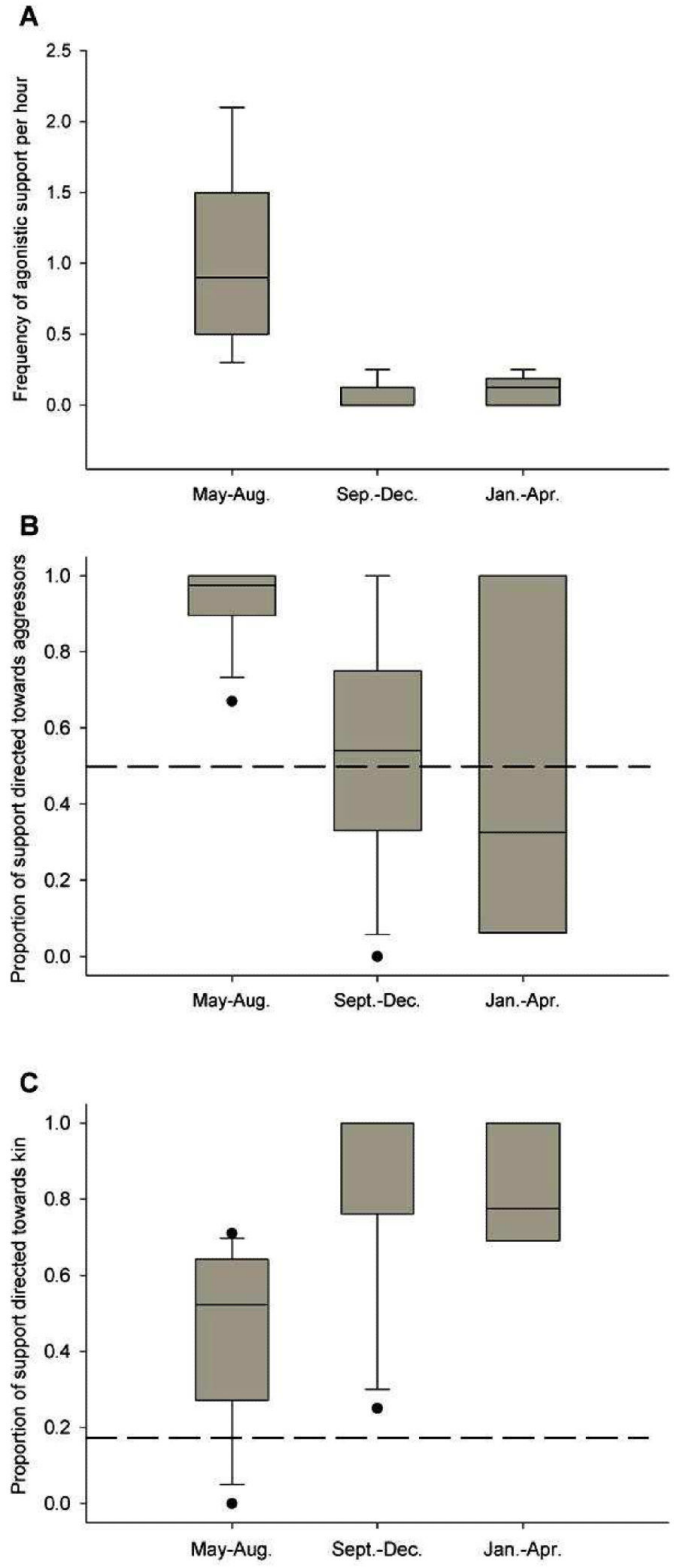
Frequency of agonistic support (**A**), proportion of aggressor support (**B**) and proportion of kin support (**C**) across developmental periods. Broken lines indicate chance levels (18.5% and 50% for kin support and aggressor support, respectively).

Agonistic support was more frequent in the first than in the second or third period (Friedman: *N* = 9, χ^2^ = 16.267, *p* < 0.001; Wilcoxon posthoc: period 1 vs. 2: *N* = 9, T = 45, *p* = 0.004; period 1 vs. 3: T = 45, *N* = 9, *p* = 0.004; period 2 vs. 3: *N* = 9, T = 4.5, *p* = 0.750, Figure 2A).

In all three periods, the subjects were more likely to support kin than non-kin (period 1: *χ^2^* = 46.664, *df* = 1*, p* < 0.001, period 2: *χ^2^* = 266.824, *df* = 1, *p* < 0.001, period 3: *χ^2^* = 222.309, *df* = 1, *p* < 0.001, Figure 2C). Nevertheless, kin support differed across periods (Friedman: *χ^2^* = 7.467, *N* = 8, *p* = 0.024), apparently increasing over time. However, no significant differences were found among pair-wise comparisons after Bonferroni corrections (all combinations, *p* > 0.05). In the first period, the subjects were more likely to support the aggressor than the victim (*χ^2^* = 75.401, *df* = 1, *p* < 0.001, Figure 2B), whereas in subsequent periods, no such effect was found, and overall the frequency of aggressor support did not differ among periods (Friedman: *χ^2^* = 4.846, *N* = 8, *p* = 0.089).

Agonistic support at the dyadic level was significantly positively correlated with allo-preening (Mantel test with 10000 permutations: *Z* = 1672955.739, *R* = 0.594, *p* = 0.003).

## Discussion

Levels of both socio-positive behaviour and severe aggression exhibited by the subjects were influenced by the developmental period, with levels of socio-positive behaviour dramatically increasing and levels of aggression showing a significant decrease following the first period. Interestingly, this shift in behaviour corresponded with the formation of a linear dominance hierarchy. An established dominance hierarchy conventionalises priority of access to competitive resources, thus avoiding repeated escalation of aggressive conflict among group members (Drews, 1993; Rowell, 1974). If a dominance hierarchy is achieved through agonistic interactions and is based on the memory of the outcomes of past encounters, individuals should be highly aggressive to one another at the beginning and decrease their aggressive behaviour when the hierarchy is stable, as shown e.g., for jungle crows (*Corvus macrorhynchos*) (Guhl, 1968; Izawa & Watanabe, 2008). Our results clearly support this prediction, as ravens showed a lower frequency of aggression after a stable dominance hierarchy formed. The DC-index also increased to 0.92, which means that nearly all forced retreat interactions were unidirectional. Note that forms of aggression in juveniles do not resemble ‘playful’ precursors of what could eventually become ‘true’ adult aggression but can result in serious injuries even as early as their first summer.

For corvids, forming a coalition with another group member may increase the social status of both participants (Emery et al., 2007; Gwinner, 1964; Lorenz, 1931). Thus a possible explanation for the increase in socio-positive behaviour from the first to the latter periods could be that the ravens were using such affiliative interactions to maintain and intensify valuable social relationships enabling the formation of such coalitions (de Waal & Luttrell, 1988; Seyfarth & Cheney, 1984). Indeed, we found a highly significant correlation between preening and agonistic support suggesting that partners who preen each are also likely to support each other in agonistic interactions against others.

Kin are more likely to share valuable relationships than non-kin because any benefits provided to kin also increases the fitness of the provider (Hamilton, 1964). Accordingly, our data show a higher frequency of socio-positive behaviour towards kin than nonkin, confirming previous findings (Fraser & Bugnyar, 2010). Such relationships may play an important role in the transfer of information among kin, leading to enhanced social learning among raven siblings (Schwab, Bugnyar, Schloegl, & Kotrschal, 2008). In spectacled parrolets (*Forpus conspicillatus*), another social bird species, strong sibling relationships are important for socialization in crèches (Wanker, 1999; Wanker, Bernate, & Franck, 1996). Interestingly, our findings are in contrast to previous studies on free living ravens that suggest no or just a minor role for kinship in raven groups (Heinrich, Kaye, & Knight, 1994; Parker, Waite, Heinrich, & Marzluff, 1994). At the moment we can only speculate why this is the case. It could be that the patterns exist in the wild but the fission-fusion dynamics of non-breeder groups make them difficult to detect; alternatively, some of our findings may be an artefact of the stable living environment of our ravens in captivity

We found the frequency of intolerance to be lower between kin than non-kin, showing that kin are more tolerant to each other and that low-intensity aggression is less likely to occur. This conforms perfectly to the assumptions of kin selection theory, predicting a lower likelihood of escalation from conflicts of interest to aggressive conflicts between kin as the costs of such escalation, and the benefits of its avoidance, are higher for kin than for non-kin (Hamilton, 1964). Interestingly, we did not find this pattern for high intensity aggression. In general, high intensity aggression occurs much less frequently than intolerant or socio-positive behaviour, so the lack of a significant effect of kinship on high-intensity aggression could be due to insufficient data.

Sex combination only appeared to influence the level of intolerance within a dyad, with females showing more intolerance towards males than males towards females. This can be explained by the fact that from November onwards all males outranked all females and therefore females were more likely to retreat from males and show defensive behaviour consistent with an intolerant relationship. Our finding that males outranked females is consistent with results from other studies on ravens (Marzluff & Heinrich, 1991) and on close relatives, such as carrion crows (*Corvus corone corone*) (Chiarati, Canestrari, Vera, Marcos, & Baglione, 2010) and jungle crows (*Corvus macrorhynchos*) (Izawa & Watanabe, 2008). These results further stress the importance of considering a dyadic relationship from both partners’ points of view, rather than considering all relationships to be symmetrical (Fraser & Bugnyar, 2010).

In the first developmental period, when there was still no linear dominance hierarchy, agonistic support was much more frequent than in the other periods. As the ravens primarily supported the aggressor during this period, coalitions appeared to be quite unstable and the ravens may simply have followed the rule of thumb “help the aggressor.” This can increase the probability of winning a fight and hence may increase social rank. A similar ‘rule of thumb’ method of choosing whom to support has been described in spotted hyenas, which always help the dominant individual in an ongoing fight, regardless of which opponent initiated the aggression (Engh et al., 2005). In playful interactions, young dogs have been reported to selectively target the ‘loser’ (Ward et al., 2009). Interestingly, the ravens appeared not to maintain their ‘rule of thumb’ strategy for long as from the beginning of the second period, the ravens were not more likely to support the aggressor than the victim. This indicates a change in behaviour towards selective support, possibly due to a progress in understanding relationships and applying this knowledge strategically to form coalitions with valuable partners. Again, kin selection appears to play an important role in coalition formation throughout development, as the ravens were more likely to support their kin across all periods.

Taken together, our results point to an important shift in interaction patterns among group members at 4-5 months post-fledging. At this age, the ravens developed a stable dominance hierarchy, became more flexible in choosing which partners to support, increased their levels of socio-positive behaviour and decreased levels of aggression. These changes coincide with changes in cognitive development associated with caching food behind objects (Bugnyar et al., 2007) and geometrical gaze following (Schloegl et al., 2007). Thus, it appears that this period may represent a critical stage in the process of mental maturation. Further understanding of this rapid cognitive shift may help us to better understand the ontogeny and the evolution of intelligence in this species. Interestingly, the critical time period of behavioural and cognitive changes roughly coincides with the time period where young ravens in the wild start integrating into non-breeder flocks (Haffer, 1993; Heinrich et al., 1994). Although this study was conducted on a single population of aviary-housed ravens, and thus caution must be taken when generalising to ravens as a whole, our findings are testament to the complexity of raven social relationships, even among juveniles, and represent an important advance in our understanding of the development of their impressive socio-cognitive abilities.

## Figures and Tables

**Table 1:** Varimax rotated component matrix

Behavioural Variables	Component		
	1	2	3
Touching	−0.071	**0.899**	0.061
Preening	0.038	**0.924**	−0.030
Contact sitting	−0.107	**0.924**	−0.050
Retreat	**0.824**	−0.065	−0.201
Forced retreat	**0.880**	−0.098	−0.141
Defensive behaviour	**0.862**	0.026	−0.124
Threat back	**0.745**	−0.043	0.375
Fighting	−0.158	−0.010	**0.934**

Values represent coefficients of correlation between each variable and each component. Values of > 0.5 or < −0.5 (marked in bold) were considered high loadings.

## References

[R1] Bergman TJ, Beehner JC, Cheney DL, Seyfarth RM (2003). Hierarchical classification by rank and kinship in baboons. Science.

[R2] Budaev S (2010). Using principal components and factor analysis in animal behaviour research: Caveats and guidelines. Ethology.

[R3] Bugnyar T, Heinrich B (2005). Ravens, *Corvus corax*, differentiate between knowledgeable and ignorant competitors. Proceedings of the Royal Society B: Biological Sciences.

[R4] Bugnyar T, Kotrschal K (2002). Observational learning and the raiding of food caches in ravens, *Corvus corax*: Is it ‘tactical’ deception?. Animal Behaviour.

[R5] Bugnyar T, Stöwe M, Heinrich B (2004). Ravens, *Corvus corax*, follow gaze direction of humans around obstacles. Proceedings of the Royal Society B: Biological Sciences.

[R6] Burnham KP, Anderson DR (2004). Multimodel inference: Understanding AIC and BIC in model selection. Sociological Methods and Research.

[R7] Byrne RW, Mellars PA, Gibson KR (1996). Relating brain size to intelligence. Modelling the early human mind.

[R8] Byrne RW, Whiten A (1988). Machiavellian intelligence: Social expertise and the evolution of intellect in monkeys, apes and humans.

[R9] Cheney DL, Seyfarth RM (1986). The recognition of social alliances by vervet monkeys. Animal Behaviour.

[R10] Cheney DL, Seyfarth RM (1989). Redirected aggression and reconciliation among vervet monkeys, *Cercopithecus aethiops*. Behaviour.

[R11] Cheney DL, Seyfarth RM (1990). How monkeys see the world: Inside the mind of another species.

[R12] Chiarati E, Canestrari D, Vera R, Marcos JM, Baglione V (2010). Linear and stable dominance hierarchies in cooperative carrion crows. Ethology.

[R13] Connor RC (2007). Dolphin social intelligence: Complex alliance relationships in bottlenose dolphins and a consideration of selective environments for extreme brain size evolution in mammals. Philosophical Transactions of the Royal Society B: Biological Sciences.

[R14] Cords M, Whiten A, Byrne RW (1997). Friendships, alliances, reciprocity and repair. Machiavellian intelligence II: Extensions and evaluations.

[R15] Cords M, Aureli F, Aureli F, de Waal FBM (2000). Reconciliation and relationship qualities. Natural conflict resolution.

[R16] de Vries H (1995). An improved test of linearity in dominance hierarchies containing unknown or tied relationships. Animal Behaviour.

[R17] de Vries H, Netto WJ, Hanegraaf PLH (1993). MatMan: A program for the analysis of sociometric matrices and behavioural transition matrices. Behaviour.

[R18] de Waal FBM, Luttrell LM (1988). Mechanisms of social reciprocity in three primate species: Symmetrical relationship characteristics or cognition?. Ethology and Sociobiology.

[R19] de Waal FBM, Tyack PL (2003). Animal social complexity: Intelligence,cCulture and individualized societies.

[R20] Drews C (1993). The concept and definition of dominance in animal behaviour. Behaviour.

[R21] Dunbar RIM (1992). Neocortex size as a constraint on group size in primates. Journal of Human Evolution.

[R22] Dunbar RIM, Bever J (1998). Neocortex size predicts group size in carnivores and some insectivores. Ethology.

[R23] Dunbar RIM, Shultz S (2007). Evolution in the social brain. Science.

[R24] Emery NJ (2006). Cognitive ornithology: the evolution of avian intelligence. Philosophical Transactions of the Royal Society B: Biological Sciences.

[R25] Emery NJ, Seed AM, Von Bayern AMP, Clayton NS (2007). Cognitive adaptions of social bonding in birds. Philosophical Transactions of the Royal Society B: Biological Sciences.

[R26] Engh AL, Siebert ER, Greenberg DA, Holekamp KE (2005). Patterns of alliance formation and postconflict aggression indicate spotted hyaenas recognize third-party relationships. Animal Behaviour.

[R27] Fraser ON, Bugnyar T (2010). The quality of social relationships in ravens. Animal Behaviour.

[R28] Fraser ON, Schino G, Aureli F (2008). Components of relationship quality in chimpanzees. Ethology.

[R29] Freeman LC, Freeman SC, Romney AK (1992). The implications of social structure for dominance hierarchies in red deer, *Cervus elaphus* L. Animal Behaviour.

[R30] Guhl AM (1968). Social inertia and social stability in chickens. Animal Behaviour.

[R31] Paz-Y-Miño GC, Bond AB, Kamil AC, Balda RP (2004). Pinyon jays use transitive inference to predict social dominance. Nature.

[R32] Gwinner E (1964). Untersuchungen über das Ausdrucks- und Sozialverhalten des Kolkraben (*Corvus corax corax* L.). Zeitschrift für Tierpsychologie.

[R33] Haffer J, Glutz von Blotzheim UN, Bauer KM (1993). *Corvus corax*: Kolkrabe. Handbuch der Vögel Mitteleuropas.

[R34] Hamilton WD (1964). Genetical evolution of social behaviour 1 & 2. Journal of Theoretical Biology.

[R35] Heinrich B, Kaye D, Knight T (1994). Dispersal and association among common ravens. The Condor.

[R36] Holm S (1979). A simple sequentially rejective multiple test procedure. Scandinavian Journal of Statistics.

[R37] Huber B (1991). Bildung, Alterszusammenseztung und Sozialstruktur von Gruppen nichtbrütender Kolkraben (*Corvus corax* L.). Metelener Schriftenreihe für Naturschutz.

[R38] Humphrey NK, Bateson PPG, Hinde RA (1976). The social function of intellect. Growing points in ethology.

[R39] Izawa E-I, Watanabe S (2008). Formation of linear dominance relationship in captive jungle crows (*Corvus macrorhynchos*): Implications for individual recognition. Behavioural Processes.

[R40] Jackson DA, Somers KM (1989). Are probability estimates from the permutation model of Mantels test stable?. Canadian Journal of Zoology.

[R41] Jolly A (1966). Lemur social behavior and primate intelligence. Science.

[R42] Judge PG (1991). Dyadic and triadic reconciliation in pigtail macaques (*Macaca nemestrina*). American Journal of Primatology.

[R43] Judge PG, Mullen SH (2005). Quadratic postconflict affiliation among bystanders in a hamadryas baboon group. Animal Behaviour.

[R44] Landau HG (1951). On dominance relations and the structure of animal societies: I. Effect of inherent characteristics. Bulletin of Mathematical Biophysics.

[R45] Lewis KP (2000). A comparative study of primate play behaviour: Implications for the study of cognition. Folia Primatologica.

[R46] Lorenz KZ (1931). Beiträge zur Ethologie sozialer Corviden. Journal für Ornithologie.

[R47] Marino L (2002). Convergence of complex cognitive abilities in cetaceans and primates. Brain, Behavior and Evolution.

[R48] Martin P, Bateson P (1993). Measuring behaviour: An introductory guide.

[R49] Marzluff JM, Heinrich B (1991). Foraging by common ravens in the presence and absence of territory holders: An experimental analysis of social foraging. Animal Behaviour.

[R50] Parker PG, Waite TA, Heinrich B, Marzluff JM (1994). Do common ravens share ephemeral food resources with kin? DNA fingerprinting evidence. Animal Behaviour.

[R51] Ratcliff D (1997). The raven.

[R52] Rowell TE (1974). The concept of social dominance. Behavioral Biology.

[R53] Sawaguchi T, Kudo H (1990). Neocortical development and social structure in primates. Primates.

[R54] Scheiber IBR, Weiß BM, Hirschenhauser K, Wascher CAF, Nedelcu IT, Kotrschal K (2008). Does relationship intelligence make big brains in birds?. Open Biology Journal.

[R55] Schloegl C, Kotrschal K, Bugnyar T (2007). Gaze following in common ravens, *Corvus corax*: Ontogeny and habituation. Animal Behaviour.

[R56] Schnell GD, Watt DJ, Douglas ME (1985). Statistical comparison of proximity matrices: Applications in animal behaviour. Animal Behaviour.

[R57] Schwab C, Bugnyar T, Schloegl C, Kotrschal K (2008). Enhanced social learning between siblings in common ravens, *Corvus corax*. Animal Behaviour.

[R58] Senar JC, Camerino M, Metcalfe NB (1990). Familiarity breeds tolernace - the developement of social stability in flocking siskins (*Carduelis spinus*). Ethology.

[R59] Seyfarth RM, Cheney DL (1984). Grooming, alliances and reciprocal altruism in vervet monkeys. Nature.

[R60] Shultz S, Dunbar RI (2006). Both social and ecological factors predict ungulate brain size. Proceedings of the Royal Society B: Biological Sciences.

[R61] Silk JB (2002). Using the ‘F’-word in primatology. Behaviour.

[R62] Sinha A (1998). Knowledge acquired and decisions made: Triadic interactions during allogrooming in wild bonnet macaques, *Macaca radiata*. Philosophical Transactions of the Royal Society B: Biological Sciences.

[R63] Tabachnick BG, Fidell LS (2007). Using multivariate statistics.

[R64] van Hooff JARAM, Wensing JAB, Frank H (1987). Dominance and its behavioural measures in a captive wolf pack. Man and wolf.

[R65] van Schaik CP, Aureli F, Aureli F, de Waal FBM (2000). The natural history of valuable relationships in primates. Natural conflict resolution.

[R66] Wanker R (1999). Socialization in spectacled parrotlets (*Forpus conspicillatus*): How juveniles compensate for the lack of siblings. Acta Ethologica.

[R67] Wanker R, Bernate LC, Franck D (1996). Socialization of spectacled parrotlets *Forpus conspicillatus*: The role of parents, crèches and sibling groups in nature. Journal für Ornithologie.

